# Density of breast: An independent risk factor for developing breast cancer, a prospective study at two premium breast centers

**DOI:** 10.1002/cam4.2821

**Published:** 2020-03-04

**Authors:** Chia Hwee Lo, Xin Ying Chai, Shirley Shy Wen Ting, Sze Chao Ang, Xinlin Chin, Lay Teng Tan, Peeroo Saania, Tuan Nur' Azmah Tuan Mat, Seniyah Mat Sikin, Anil Gandhi

**Affiliations:** ^1^ Jeffrey Cheah School of Medicine and Health Sciences Monash University Bandar Sunway Malaysia; ^2^ Department of Surgery Hospital Sultanah Aminah (HSA) Johor Bahru Malaysia; ^3^ Department of Surgery Hospital Sultan Ismail (HSI) Johor Bahru Malaysia

**Keywords:** BI-RADS, breast cancer, breast density

## Abstract

**Background:**

Breast cancer is the leading cause of death among women worldwide. Studies have identified breast density as a controversial risk factor of breast cancer. Moreover, studies found that breast density reduction through Tamoxifen could reduce risk of breast cancer significantly. To date, no study on the association between breast density and breast cancer has been carried out in Malaysia. If breast density is proven to be a risk factor of breast cancer, intervention could be carried out to reduce breast cancer risk through breast density reduction.

**Purpose:**

To determine if density of breast is an independent risk factor which will contribute to development of breast cancer.

**Materials and Methods:**

A prospective cohort study is carried out in two hospitals targeting adult female patients who presented to the Breast Clinic with symptoms suspicious of breast cancer. Participants recruited were investigated for breast cancer based on their symptoms. Breast density assessed from mammogram was correlated with tissue biopsy results and final diagnosis of benign or malignant breast disease.

**Results:**

Participants with dense breasts showed 29% increased risk of breast cancer when compared to those with almost entirely fatty breasts (odds ratio [OR] 1.29, 95% CI 0.38‐4.44, *P* = .683). Among the postmenopausal women, those with dense breasts were 3.1 times more likely to develop breast cancer compared with those with fatty breasts (OR 3.125, 95% CI 0.72‐13.64, *P* = .13). Moreover, the chance of developing breast cancer increases with age (OR 1.046, 95% CI 1.003‐1.090, *P* < .05). In contrast, the density of breast decreases with increasing age (*P* < .05) and body mass index (*P* = .051). The proportion of high breast density whether in the whole sample size, premenopausal, or postmenopausal group was consistently high.

**Conclusion:**

Although results were not statistically significant, important association between breast density and risk of breast cancer cannot be ruled out. The study is limited by a small sample size and subjective assessment of breast density. More studies are required to reconcile the differences between studies of contrasting evidence.

## Graphical abstract

There are many established risk factors of breast cancer, and density of breast is a controversial risk factor which we wanted to evaluate. Our study shows that postmenopausal women with dense breasts are 3 times more likely to develop breast cancer compared to those with low breast density.

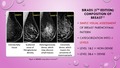

## 1 INTRODUCTION

In Malaysia, breast cancer implicates a tremendous health risk. The Malaysian National Cancer Registry Report states that breast cancer is the most common cancer among Malaysian women.^1^ From 2007 to 2011, a total of 18,206 breast cancer cases were reported. This accounted for 32.1% of all cancer cases among Malaysian females. Breast cancer had peak incidence in the 50‐59 age group. There are many established risk factors of breast cancer namely, age, obesity, and family history. Our topic of interest is a particularly controversial risk factor, that is, density of breast.

Breast density is defined as the relative amount of fibroglandular tissue as compared with fatty breast tissue seen on mammogram.^2^ There is much ambiguity when referring to high breast density as a cancer risk because its significance is highly debatable. Some authors argue that the association between breast density and breast cancer is confounded by other risk factors and masking effect of high breast density. Nevertheless, current literature asserts that there is a meaningful relationship between high density of breast and breast cancer. Breast cancer most commonly arises from the epithelial elements of the breast and it is the amount of these elements that determines its density.^2^ Thus, it is possible for breast density to influence breast cancer risk.

High breast density is known to decrease sensitivity of mammography by obscuring small masses or lesions. A large cohort study in the United States remarked a decline in sensitivity of film‐screen mammography from 88.8% in patients with almost entirely fatty breasts to 68.1% in patients whose breasts are extremely dense.^3^

A systematic review of 42 studies conducted by McCormack and Silva in 2006 described a 4.64‐fold increased risk of breast cancer among women with ≥75% breast density (RR 4.64, 95% CI 3.64‐5.91) in contrast to those with <5% dense area.^4^ Even after excluding interval cancers, this strong association persisted, indicating the risk is not entirely explained by masking bias. On the other hand, Vierkant^5^ found that breast density may not play a role in risk estimation of breast cancer among those with atypical hyperplasia.

Density of breast has shown great potential in risk modification through the modality of chemoprevention. Cuzick found that with ≥10% reduction in mammographic density within 12‐18 months, breast cancer was significantly reduced by 63%.^6^

To date, no study on the association between breast density and breast cancer has been carried out in Malaysia. There is a need to raise awareness regarding the potential of breast density as a risk factor so that women can make informed health decisions. This study attempts to assess the degree to which high breast density poses as a cancer risk in the local setting.

## 2 MATERIALS AND METHODS

### 2.1 Ethics

Ethical approval was obtained from Medical Research and Ethics Committee's, Ministry of Health, Malaysia and Monash University Human Research Ethics Committee before the start of study.

### 2.2 Study design

This is a prospective cohort study done in Hospital Sultanah Aminah and Hospital Sultan Ismail, Johor, Malaysia. The population targeted was adult female patients who presented to the Breast Clinic with symptoms suspicious of breast cancer. Inclusion criteria of subjects encompassed female gender, aged 18 years and above, ability to give informed consent, symptomatic, and would be undergoing mammography as well as tissue biopsy. Exclusion criteria comprised of male patients, those who withdrew consent or were lost to follow up, patients who underwent breast ultrasound only, those who underwent either mammography or tissue biopsy only, patients with primary cancer of other sites, those with previous history of breast cancer, and those who had undergone breast surgery.

Under demographics, data on age, date of birth, race, height, and weight were collected. Information including parity, family history, age at menarche, menopausal status, and usage of oral contraceptive pill (OCP) or harmonal replacement therapy (HRT) were also noted. Breast density of patients was obtained from the mammography report as categorized using BI‐RADS composition of breast. The outcome of each participant was the diagnosis of either benign or malignant breast disease. A total of 174 participants were recruited but only 107 participants met the inclusion criteria.

### 2.3 Sample size

Sample size for this study was calculated using Select statistical services.^7^ Relative precision was set at 95%. A margin error of 5% and confidence level of 95% were applied. According to a local study on the prevalence of breast density, 60% of Malaysian women have dense breasts.^8^ Based on this, the ratio of ‘dense’ to ‘not dense’ was set at 1.5. The percentage of women with less dense breasts found to have breast cancer was estimated to be half of those with dense breasts. The expected odds ratio of dense breast causing breast cancer was set at 4, based on a study done by McCormack.^4^ With that, the statistical calculator estimated that a sample size of 101 was needed to achieve statistical significance.

### 2.4 Statistical analyses

Statistical Package for the Social Sciences version 25.0 was used to conduct statistical analyses. The Kolmogorov‐Smirnov test was applied to quantitative variables to test for normality of the distribution. Descriptive data were expressed in mean and standard deviation. If data were skewed, median and interquartile range were used instead. Pearson Chi‐square test was conducted for test of association. Chi‐square test was used to test for homogeneity for categorical data. Whenever assumptions were not met, Fisher's exact test was used. For risk analysis, bivariate logistic regression was conducted for every single factor associated with breast cancer. Results yielded were expressed in odds ratio (OR) and those with *P* value of <.05 were considered statistically significant.

## 3 RESULTS

Of 107 participants, more than half (n = 58) were diagnosed with breast cancer. This translated to 54% cancer cases and 46% benign cases.

In this group of participants, dense breasts were found to be more prevalent. As high as 66% of them have BI‐RADS C ‘heterogeneously dense’ breasts. This is followed by BI‐RADS B and BI‐RADS A with 22% and 11% of participants, respectively. Only 1 participant fell under BI‐RADS D ‘extremely dense’ category. As a result, BI‐RADS C and BI‐RADS D were combined into a single category termed as ‘dense’ breasts for the purpose of statistical analyses in this study.

Table 1 displays the demographics and characteristics of the study participants. The median age in this group is 52 years old with a range 48‐63 years old. Most of them are of Malay ethnicity (57%), followed by Chinese (30%) and Indians (12%). Nearly 60% of the participants belong to the ‘overweight’ and ‘obese’ body mass index (BMI) groups with a median BMI of 26.4 kg/m^2^. Of 107, 48 (45%) of them are premenopausal, whereas 59 (55%) have attained menopause. In addition, more than 85% of the group did not report family history of breast cancer and significant use of hormonal therapy (more than 5 years). Nearly all attained menarche at the age of 12 and above. Fifty‐four percentage of the women have at least 3 children, whereas 8% are nulliparous.

**Table 1:** Distribution of participant demographics and risk factors

Risk Factor	Overall n (%)	Median (range)
Age (y)		52 (48‐63)
Race		
Malay	61 (57)	
Chinese	32 (30)	
Indian	13 (12)	
Others	1 (1)	
BMI (kg/m^2^)		26.4 (23.0‐29.4)
<18.5	7 (6.5)	
18.5‐24.9	37 (34.6)	
25.0‐29.9	38 (35.5)	
≥30.0	25 (23.4)	
Menopausal status		
Premenopausal	48 (44.9)	
Postmenopausal	59 (55.1)	
Family history of breast cancer		
No	91 (85)	
Yes	16 (15)	
Use of OCP/HRT		
No	94 (87.9)	
Yes	13 (12.1)	
Age at menarche		13 (12‐15)
<12	4 (3.7)	
≥12	103 (96.3)	
Parity		3 (2‐4)
Nulliparous	9 (8.4)	
1‐2	40 (37.4)	
≥3	58 (54.2)	
Breast density (BI‐RADS)		
A – almost entirely fatty	12 (11.2)	
B – scattered fibroglandular density	24 (22.4)	
C – dense (heterogeneously and extremely)	71 (66.4)	
Total	107 (100)	

Abbreviations: HRT, harmonal replacement therapy; OCP, oral contraceptive pill.

Table 2 shows the findings of relationship analysis between various known risk factors of breast cancer and different categories of breast density. Participants in the three categories of breast density varied significantly in terms of age and menopausal status. Age was inversely associated with breast density. The mean age in the fatty breasts group decreases from 62 years to a median age of 51 years in the dense breasts category (*P* < .05). In the BI‐RADS A group, the proportion of postmenopausal women was 83.3%, whereas 16.7% were premenopausal. In contrast, a higher percentage of premenopausal women (53.5%) were seen in BI‐RADS C group compared to 46.5% of those who have achieved menopause (*P* < .05). BMI was found to be 30.4 kg/m^2^ in the lowest density group and 26.2 kg/m^2^ in the highest density category (*P* = .051). The highest percentage of dense breasts was seen among Chinese women (72%, 23 of 32) followed by 66% in Malay and 54% in Indian women (*P* = .265). Factors such as parity, family history, age of menarche, and hormone therapy use were not significantly associated with BI‐RADS density measurement.

**Table 2:** Association between known risk factors and breast cancer

BI‐RADS Category				
Characteristics	A—Fatty Mean (SD)	B—Scattered Mean (SD)	C—Dense Median (range)	*P* value
Age (y)	62 (8)	57 (9.8)	51 (47‐59)	<.05
BMI (kg/m^2^)	30.4 (5.7)	25.8 (4.9)	26.2 (23‐29.4)	.051
	n (%)	n (%)	n (%)	
Race				.265
Malay	7 (58.3)	14 (58.3)	40 (56.3)	
Chinese	1 (8.3)	8 (33.3)	23 (32.4)	
Indian	4 (33.3)	2 (8.3)	7 (9.9)	
Others	0 (0.0)	0 (0.0)	1 (1.4)	
Menopausal status				<.05
Premenopausal	2 (16.7)	8 (33.3)	38 (53.5)	
Postmenopausal	10 (83.3)	16 (66.7)	33 (46.5)	
Family history				.543
No	9 (75)	21 (87.5)	61 (85.9)	
Yes	3 (25)	3 (12.5)	10 (14.1)	
Use of OCP/HRT				.816
No	10 (83.3)	21 (87.5)	63 (88.7)	
Yes	2 (16.7)	3 (12.5)	8 (11.3)	
Age at menarche				.407
<12	0 (0.0)	2 (8.3)	2 (2.8)	
≥12	12 (100)	22 (91.7)	69 (97.2)	
Parity				.617
Nulliparous	0 (0.0)	2 (8.3)		
1‐2	3 (25.0)	8 (33.3)		
≥3	9 (75.0)	14 (58.3)		

Abbreviations: HRT, harmonal replacement therapy; OCP, oral contraceptive pill; SD, standard deviation.

### 3.1 Association between BI‐RADS classification of breast density and breast cancer risk

No statistically significant association is found between BI‐RADS breast density categories and risk of developing breast cancer (*P* = .824). Sixty‐nine percentage of study participants diagnosed with breast cancer have dense breasts (BI‐RADS C and D).

There are equal numbers of benign and malignant cases in the low breast density groups, BI‐RADS A and B. In the high breast density group, a higher number were diagnosed with breast cancer compared to those with benign breast disease (40 vs 31).

Table 3 shows that when a woman ages by 1 year, there is 4.6% higher chance of developing breast cancer (95% CI 1.003‐1.090, *P* < .05). When comparing BI‐RADS C to BI‐RADS A, women with dense breasts were 1.29 times more likely to develop breast cancer (95% CI 0.38‐4.44, *P* = .683).

**Table 3:** Factors associated with breast cancer (n = 107)

Risk factor	Crude odds ratio (95% CI)	*P* value
Age	1.046 (1.003, 1.090)	<.05*
Race		
Malay	1 (referent)	
Chinese	0.61 (0.26, 1.45)	
Indian	0.81 (0.24, 2.70)	.265
Others	0 (0.0)	.732
BMI	1.006 (0.94, 1.07)	1.000
Age at menarche	1.14 (0.90, 1.44)	.866
Parity	1.026 (0.84, 1.25)	.275
Family history of breast cancer	1.102 (0.38, 3.21)	.799
Use of OCP/HRT	0.692 (0.22, 2.22)	.859
Breast density (BI‐RADS)		.536
A—almost entirely fatty	1 (referent)	
B—scattered fibroglandular density	1.00 (0.25, 3.99)	1.000
C—dense (heterogeneously and extremely)	1.29 (0.38, 4.44)	.683

Note

Bivariate logistic regression conducted for each risk factor.

Abbreviations: CI, confidence interval; HRT, harmonal replacement therapy; OCP, oral contraceptive pill.

* *P* value significant at <.05.

### 3.2 Subgroup analysis according to menopausal status

In the premenopausal group, no significant association was found between density of breast and development of breast cancer (*P* = .321), as shown in Table 4. Among those diagnosed with malignancy, 88.2% had dense breasts.

**Table 4:** Association between breast density and breast cancer among pre‐ and postmenopausal women (n = 48)

	Diagnosis		*P* value
	Benign	Malignant	
Premenopausal			
BI‐RADS composition of breast			.321
A—almost entirely fatty	1 (3.2)	1 (5.9)	
B—scattered fibroglandular density	7 (22.6)	1 (5.9)	
C—dense (heterogeneously and extremely)	23 (74.2)	15 (88.2)	
Postmenopausal			
BI‐RADS Composition of Breast			.295
A—almost entirely fatty	5 (27.8)	5 (12.2)	
B—scattered fibroglandular density	5 (27.8)	11 (26.8)	
C—dense (heterogeneously & extremely)	8 (44.4)	25 (61.0)	

Note

Fisher's exact test used.

There is no statistically significant association between density of breast and breast cancer risk among postmenopausal women (*P* = .295). A large proportion of those diagnosed with breast cancer have dense breasts (61%). This is in contrast with 44.4% of benign cases having high breast density.

From Table 5, it was shown that among postmenopausal women, those in BI‐RADS B group have 2.2 times the chance of developing breast cancer compared with those in BI‐RADS A (OR 2.2, 95% CI 0.43‐11.22, *P* = .343). Women with dense breasts were 3.1 times more likely to develop breast cancer compared to those with fatty breasts (OR 3.125, 95% CI 0.72‐13.64, *P* = .13).

**Table 5:** Risk of developing breast cancer according to breast density category

	Crude OR (95% CI)	*P* value
Premenopausal		
BI‐RADS composition of breast		
A—almost entirely fatty	1 (referent)	
B—scattered fibroglandular density	0.143 (0.004, 4.61)	.272
C—dense (heterogeneously and extremely)	0.652 (0.04, 11.24)	.769
Postmenopausal		
BI‐RADS composition of breast		
A—almost entirely fatty	1 (referent)	
B—scattered fibroglandular density	2.200 (0.43, 11.22)	.343
C—dense (heterogeneously and extremely)	3.125 (0.72, 13.64)	.130

Note

Bivariate logistic regression applied.

Abbreviations: CI, confidence interval; OR, odds ratio.

## 4 DISCUSSION

After conducting a thorough literature search in this field, it is believed that this study is the first in Malaysia to correlate density of breast with breast cancer. It is hypothesized that Malaysian women with higher breast density have a higher risk of developing breast cancer. The primary aim of this study is to determine if we can postulate whether breast density is an independent risk factor of breast cancer, in the hopes of aiding prevention and early detection of breast cancer. Results of this study indicated that participants with dense breasts have a 29% increased odds of breast cancer when compared to those with almost entirely fatty breasts (OR 1.29, 95% CI 0.38‐4.44, *P* = .683). Among postmenopausal women, those with dense breasts were 3.1 times more likely to develop breast cancer compared to those with fatty breasts (OR 3.125, 95% CI 0.72‐13.64, *P* = .13). However, it is noted that these analyses are not of statistical significance. Other than that, it is revealed that the chance of developing breast cancer increases with age (OR 1.046, 95% CI 1.003‐1.090, *P* < .05). In contrast, the density of breast decreases with increasing age (*P* < .05) and BMI (*P* = .051). The proportion of high breast density whether in the whole sample size, premenopausal, or postmenopausal group was consistently high. Chinese women have also shown the highest percentage of dense breasts.

The primary results of this study revealed a 1.29‐fold increased risk of breast cancer among women with BI‐RADS C density compared with BI‐RADS A category, however, it was not statistically significant. Two studies echoed similar findings as this study.^5^, ^9^ However, most literature revealed strong association, with 2 to 4 times increased risk of cancer with increasing density.^4^, ^10^ The main reason behind the difference in results yielded lies in the small sample size. Moreover, existing literature commonly utilized percent density, quantified by computational software, as point of comparison. This differs from BI‐RADS qualitative measurement of breast density used in this study which is subjective to reader's assessment. Not only that, a meta‐analysis of 42 studies revealed heterogeneity attributed to combination of different density assessment methods in single studies despite demonstrating statistically significant results.^4^ This study also focuses on the symptomatic population which may explain the differing results from available studies. A European cohort study whose sample size was the general population who underwent breast cancer screening produced statistically significant results. As most studies were conducted on Caucasian populations, variation in underlying population risk and demographics could also explain the contradicting results. Contrasting evidence exists even among available literature which justifies the need for further research in this area. As a small sample size can strongly undermine the validity of the results, there is a need for the continuation of this study.

Further analysis of the postmenopausal subgroup displayed a 3‐fold higher risk of breast cancer in women with high breast density compared with those having low breast density (*P* = .13). This shows a similar size effect to a systematic review conducted by Cummings on postmenopausal women.^11^ Authors concluded that there was 4 times cancer risk increase regardless of density measurement methods. Although the results of this study did not show statistical significance, the relative risks show a moderate size effect which may be important in clinical significance.

This study has also shown an inverse association between age and density of breast. This is consistent with results of a large cohort study in Croatia.^10^ As age increases, there is a significant decrease in breast density (*P* < .05). On the other hand, findings also demonstrate a linear relationship between age and risk of breast cancer (*P* < .05). This result corresponds to studies which have shown that age is a risk factor. Breast cancer is most prevalent among postmenopausal women who should be experiencing a reduction in breast density according to results above. This weakens the argument that high density of breast is linked with breast cancer. However, it is postulated that these post‐menopausal women with breast cancer may still have breasts denser than the average disease‐free postmenopausal women. In this study, a larger proportion of post‐menopausal women with breast malignancy was shown to have dense breasts. Sixty‐one percentage of those with breast cancer have heterogeneously and extremely dense breasts compared to 44% of the disease‐free women having similar breast density. This finding is further supported by a study that demonstrated significant increase in risk of breast cancer with increasing breast density among postmenopausal women.^12^ Although results of this study were not significant, an association between density of breast and risk of cancer still cannot be ruled out.

The interaction between breast density and known risk factors of breast cancer is further studied. Findings of this study indicate that BMI may be inversely associated with breast density. BMI of the study participants showed a downward trend while the breast density increased (*P* = .051). A large retrospective cohort study by Tesic reported similar trend of association between these two variables.^10^ The borderline significance of this study results may be attributed to the unequal distribution of BMI among the participants. There are a substantial proportion of women (60%) who are overweight and obese according to their BMI. This coupled with high proportion of dense breasts among participants may have affected the analysis. A smaller number of participants with a certain characteristic may affect the ability to identify important association. In short, the discrepancies between the results of this study and existing evidence can mostly be attributed to different methods of measuring breast density, different study population, and study design.

This study is limited by its small sample size of 107 participants. This is attributed to a short period of time for recruiting participants and collecting data. A small sample size may lead to bigger margin for errors and underestimate any association and risk effect. Therefore, validity of data analysis may have been affected. Another limitation is the qualitative measurement of breast density using BI‐RADS classification. This mode of assessment is subject to individual judgment of each radiologist. Moreover, studies have shown that it only has moderate interrater agreement.^13^ As the mammographs were read by a few radiologists in this study, accuracy of density measurement may have been affected. Besides, the focus of this study is on the symptomatic population rather than general population undergoing screening. Thus, interval cancers cannot be determined and masking bias cannot be ruled out.

Future directions of this study include continuing the study to achieve a larger sample size that meets the number estimated by the sample size calculator. Hence, this study is still ongoing thus far. Besides that, a designated radiologist should be assigned to assess the mammograms of participants in future studies to ensure consistency of readings.

The results of this study further emphasize on the ambiguity that surrounds density of breast as an independent risk factor of breast cancer. There are two implications of the findings of this study. One, there may not be an important association between these two variables. This study will then serve as a reminder to critically appraise evidence before eagerly labeling breast density as a new risk factor. Two, important associations were missed during data analyses due to under sampling of study group. Both scenarios warrant the need for further studies to obtain strong and conclusive evidence. More attention should be paid to breast density by the local medical community as evidenced by international events such as advocacy group, laws mandating the reporting of breast density, and FDA‐approved trials for reducing breast density using tamoxifen. When more studies present conclusive evidence that supports breast density as a risk factor, evidence‐based screening and prevention strategies can then be implemented to modify the risk of Malaysian women developing breast cancer.

## 5 CONCLUSION

This study was conducted to examine the hypothesis that density of breast could be an independent risk factor of breast cancer. Although results were not statistically significant, important association between breast density and cancer risk cannot be ruled out. The study shows that postmenopausal women with dense breasts are 3 times more likely to develop breast cancer compared to those with low breast density. Future studies should utilize standardized ways of measuring breast density to ensure accuracy of assessment. More studies are required to reconcile the differences between studies of contrasting evidence. Identifying factors that influence the risk of developing breast cancer can bring about great impact especially in modifying the risk of women who are going to develop breast cancer in their lifetime. Given the high prevalence of dense breasts in Malaysia, the healthcare community should investigate the clinical significance of breast density and consider its integration into future screening and prevention policies.

## CONFLICTS OF INTEREST

None.

## AUTHOR CONTRIBUTIONS

Chia Hwee Lo: Data curation, formal analysis, original draft; Xin Ying Chai: Formal analysis, review and editing; Shirley Shy Wen Ting: Data curation; Sze Chao Ang: Data curation; Xinlin Chin: Data curation; Lay Teng Tan: Data curation; Saania Peeroo: Data curation; Tuan Nur' Azmah binti Tuan Mat: Project administration, supervision; Seniyah binti Mat Sikin: Project administration, supervision; Anil Gandhi: Conceptualization, project administration, methodology, resources, formal analysis, original draft, review & editing, supervision.
